# Effects of weight change on apolipoprotein B-containing emerging atherosclerotic cardiovascular disease (ASCVD) risk factors

**DOI:** 10.1186/s12944-019-1094-4

**Published:** 2019-07-17

**Authors:** Michael L. Dansinger, Paul T. Williams, H. Robert Superko, Ernst J. Schaefer

**Affiliations:** 1 Boston Heart Diagnostics, 175 Crossing Boulevard, Suite 100, Framingham, MA 01702 USA; 2 Tufts Medical Center, 800 Washington St, Boston, MA 02111 USA; 3 Cardiovascular Nutrition Laboratory, USDA Human Nutrition Research Center at Tufts University, 711 Washington St., Boston, MA 02111 USA

**Keywords:** Cholesterol, Triglyceides, Low-density lipoproteins, Apolipoprotein B, lipoprotein(a), Weight loss, Obesity

## Abstract

**Background and aims:**

Non-high-density (HDL)-cholesterol, low-density lipoprotein (LDL)-particle number, apolipoprotein B, lipoprotein(a) (Lp(a)), and small-dense (sdLDL) and large-buoyant (lbLDL) LDL-subfractions are emerging apo B-containing atherosclerotic cardiovascular disease (ASCVD) risk factors. Current guidelines emphasize lifestyle, including weight loss, for ASCVD risk management. Whether weight change affects these emerging risk factors beyond that predicted by traditional triglyceride and LDL-cholesterol measurements remains to be determined.

**Method:**

Regression analyses of fasting ∆apo B-containing lipoproteins vs. ∆BMI were examined in a large anonymized clinical laboratory database of 33,165 subjects who did not report use of lipid-lowering medications. Regression slopes (±SE) were estimated as: *∆mmol/L per ∆kg/m^2^, ^†^∆g/L per ∆kg/m^2^, ^‡^∆% per ∆kg/m^2^, and ^§^∆μmol/L per ∆kg/m^2^.

**Results:**

When adjusted for age, ∆BMI was significantly related to ∆nonHDL-cholesterol (males: 0.0238 ± 0.0041, *P* = 7.9 × 10^− 9^; females: 0.0330 ± 0.0037, *P* < 10^− 16^)*, ∆LDL-particles (males: 0.0128 ± 0.0024, *P* = 2.1 × 10^− 7^; females: 0.0114 ± 0.0022, *P* = 3.2 × 10^− 7^)^*^, ∆apo B (males: 0.0053 ± 0.0010, *P* = 7.9 × 10^− 8^; females: 0.0073 ± 0.0009, *P* = 2.2 × 10^− 16^)^†^, ∆sdLDL (males: 0.0125 ± 0.0015, *P* = 2.2 × 10^− 16^; females: 0.0128 ± 0.0012, *P* < 10^− 16^)*, ∆percent LDL carried on small dense particles (%sdLDL, males: 0.296 ± 0.035, *P* < 10^− 16^; females: 0.221 ± 0.023, *P* < 10^− 16^)^‡^, ∆triglycerides (males: 0.0358 ± 0.0049, *P* = 2.0 × 10^− 13^; females: 0.0304 ± 0.0029, *P* < 10^− 16^)*, and ∆LDL-cholesterol (males: 0.0128 ± 0.0034, *P* = 0.0002; females: 0.0232 ± 0.0031, *P* = 1.2 × 10^− 13^)* in both males and females. Age-adjusted ∆BMI was significantly related to ∆lbLDL in females (0.0098 ± 0.0024, *P* = 3.9 × 10^− 5^)* but not males (0.0007 ± 0.0026, *P* = 0.78)*. Female showed significantly greater increases in ∆LDL-cholesterol (*P* = 0.02) and ∆lbLDL (*P* = 0.008) per ∆BMI than males. ∆BMI had a greater effect on ∆LDL-cholesterol measured directly than indirect estimate of ∆LDL-cholesterol from the Friedewald equation. When sexes were combined and adjusted for age, sex, ∆triglycerides and ∆LDL-cholesterol, ∆BMI retained residual associations with ∆nonHDL-cholesterol (0.0019 ± 0.0009, *P* = 0.03)*, ∆LDL-particles (0.0032 ± 0.0010, *P* = 0.001)*, ∆apo B (0.0010 ± 0.0003, *P* = 0.0008)^†^, ∆Lp(a) (− 0.0091 ± 0.0021, *P* = 1.2 × 10^− 5^)^§^, ∆sdLDL (0.0001 ± 0.0000, *P* = 1.6 × 10^− 11^)^*^ and ∆%sdLDL (0.151 ± 0.018, *P* < 10^− 16^) ^‡^.

**Conclusions:**

Emerging apo B-containing risk factors show associations with weight change beyond those explained by the more traditional triglyceride and LDL-cholesterol measurements.

**Electronic supplementary material:**

The online version of this article (10.1186/s12944-019-1094-4) contains supplementary material, which is available to authorized users.

## Background

Lipoprotein management has historically focused on indirect low-density lipoprotein (LDL) and triglyceride concentrations [1]. However, many individuals develop ischemic heart disease despite having had indirect LDL-cholesterols within the normal range [1, 2]. Indirect LDL-cholesterol values are calculated using the Friedewald equation [3], which is subject to inaccuracy in the presence of high triglycerides and other conditions [4]. More recently, the desire for greater accuracy has lead to the development of direct measurements of LDL-cholesterol [4]. Triglycerides are not thought to be atherosclerotic per se, but reflect high cholesterol concentrations in triglyceride-rich lipoproteins, and influence the metabolism of other lipoproteins more directly involved in the atherogenic process [1]. Current treatment guidelines do not target triglycerides concentrations under 500 mg/dL [1, 5].

Several emerging apolipoprotein (apo)-B containing risk factors have been identified due to their stronger relationships to atherosclerotic cardiovascular disease (ASCVD) risk than LDL-cholesterol, or their residual risk after adjusting for LDL-cholesterol and triglycerides. Non-high density lipoprotein (HDL) cholesterol provides a single global index of cholesterol in all apoB containing atherogenic lipoproteins, including very-low-density lipoprotein (VLDL) and triglyceride-rich remnant lipoproteins [5]. Prospectively, non-HDL-cholesterol has been shown to be superior to LDL-cholesterol in predicting increased CHD risk [6, 7], and in predicting CHD risk independent of LDL-cholesterol [8]. In clinical trials, on-study nonHDL-cholesterol was a better predictor of CVD-risk than LDL-cholesterol in the treating to new targets (TNT) and Incremental Decrease in End Points Through Aggressive Lipid Lowering (IDEAL) trials [9], and was closely associated with coronary atheroma progression irrespective of on-study LDL-cholesterol [10]. It has been identified as a secondary target when triglycerides ≥200 mg/dL [1].

Apolipoprotein (apo) B concentrations measure total LDL, intermediate density lipoproteins (IDL), very low density lipoproteins (VLDL), and lipoprotein(a) (Lp(a)) particle concentrations because each particle contains exactly one apoB100 molecule [1]. Prospectively, it has been shown to improve risk prediction for cardiovascular events after controlling for traditional risk factors [11]. Clinical trials show that on-study apo B concentrations were better predictors of CVD-risk than LDL-cholesterol [9]. Apo B concentrations of < 90 mg/dL (< 80 in very high risk patients) is the target goal proposed as by National Lipid Association in patients treated for cholesterol [2].

ASCVD is thought to be more a function of the total number of apo B containing particles than LDL-cholesterol, which is not a reliable estimate of LDL particle number. LDL-particle concentrations show a stronger association with future CVD risk than either LDL-cholesterol or non HDL-cholesterol, and in the case of discordant LDL-particle and LDL-concentrations, CVD risk tracks with LDL-particle and not LDL-cholesterol [12, 13]. Finally, we note that LDL is made up of multiple particles subclasses that differ by size, density and composition [14]. Smaller-denser LDL particles measured by their cholesterol concentration (sdLDL) or as a percentage of the total LDL-cholesterol (%sdLDL) have been associated with increased ASCVD risk, whereas there is much less evidence linking larger buoyant LDL (lbLDL) to increased risk [15].

Weight loss and other lifestyle modifications play a major role in managing dyslipidemia [16]. A three-kg weight loss reduces triglyceride concentrations by at least 0.17 mmol/L (15 mg/dL), and a 5- to 8-kg weight loss produces a 0.13 mmol/L (5-mg/dL) average LDL-cholesterol reduction [16]. There is a manifest need to identify the effects of weight loss on emerging ASCVD risk factors, and to assess whether they differs from their expected effects given their associations with triglycerides and LDL-cholesterol. To this end, we analyzed the relationships between changes (∆) in clinically reported weight and ∆plasma concentrations of apo B-containing risk factors in a national anonymized clinical laboratory database. The relationships of ∆plasma HDL-subclass concentrations and ∆BMI have been previously reported [17].

## Methods

Epidemiological analyses of the large clinical laboratory datasets have been previously used in the study of the effects of health policy [18], environmental impact [19], temporal trends [20, 21], lipoproteins [22, 23], and other blood components [24] on health biomarkers. There were 67,210 women and 57,375 men not on lipid-lowering medications whose physicians provided clinical BMI measurements and sent fasting blood samples to Boston Heart Diagnostics for laboratory analysis between December 16, 2010 and October 31, 2017. Age, gender, height, weight, and use of lipid-lowering medications were obtained from the sample submission form. Laboratory assays of the blood draws were made at the time of the blood draws for total cholesterol (enzymatic colorimetric), triglycerides (enzymatic colorimetric), low-density lipoprotein (LDL)-cholesterol (direct, enzymatic colorimetric), and HDL-cholesterol (enzymatic colorimetric). All analyses were performed on anonymized data collected in a large clinical laboratory and are exempt from human subjects. The dataset has been previously reported in our studies of changes in weight vs. HDL-subfractions [17] and temporal changes in LDL-cholesterol concentrations following the promotion of the treat-to-risk guidelines for cholesterol management [25].

Indirect LDL-cholesterol was obtained from the Friedewald equation: total cholesterol – triglycerides/5 for triglycerides ≤400 mg/dL and direct LDL-cholesterol for triglycerides > 400. [3] Direct LDL-cholesterol and sdLDL-cholesterol were measured using automated standardized enzymatic analysis on a Hitachi 911 automated analyzer and kits provided by the Denka Seiken Corporation, Tokyo, Japan. Specifically, we incubated plasma (0.1 ml) combined with 0.1 ml precipitation reagent containing heparin sodium salt and MgCl for 10 min at 37 °C. sdLDL-C was then measured by the homogenous method after separation of the sdLDL fraction (d = 1.044–1.063 g/ml) and HDL by filtration [26–29]. Large buoyant LDL (lbLDL)-cholesterol values were calculated as difference between direct LDL-cholesterol – sdLDL-cholesterol. The percentage of total LDL-cholesterol carried on the small dense LDL (%sdLDL-cholesterol) was calculated as 100*sdLDL-cholesterol/total LDL-cholesterol. Lp(a) levels using the Denka Seiken latex-enhanced turbidimetric immunoassay. LDL-particle concentrations were determined by nuclear magnetic resonance (NMR), and apo B concentrations by immunoturbidimetric method.

### Statistical analyses

Statistical analyses of the anonymized data were performed using the JMP statistical package for the Macintosh (version 13, SAS institute, Cary, NC). Results are presented as means (standard deviations) and slopes ±SE. Subjects who reported the use of cholesterol-lowering medications were excluded. Age-adjustment was achieved using sex-specific second-order polynomial of age and age^2^. Results are presented in Système international (SI) units with the conventional units provided as Additional file 1. The first follow-up visit is considered the primary due to its substantially larger sample size than the second and third follow-up visits.

## Results

At baseline, the men averaged 58.4 (13.7) and the women 58.1 (14.0) years of age. Their baseline body weight distribution were: 0.3% of men and 1.4% of women were underweight (BMI < 18.5 kg/m^2^), 17.6% of men and 32.5% of women were healthy weight (18.5 ≤ BMI < 25 kg/m^2^); 41.4% of men and 30.4% of women were overweight (25 ≤ BMI < 30 kg/m^2^); 25.9% of men and 18.8% of women were class I obese (30 ≤ BMI < 35 kg/m^2^); 9.6% of men and 9.7% of women were class II obese (35 ≤ BMI < 40 kg/m^2^); and 5.1% of men and 7.2% of women were class III obese (BMI > 40 kg/m^2^). The first, second, and third follow-up visits averaged 0.8, 1.4, and 1.8 years after baseline, respectively. The men lost an average of − 0.12 (2.02) kg/m^2^ by the first follow-up, − 0.21 (2.13) kg/m^2^ by the second follow-up, and − 0.22 (2.36) kg/m^2^ by the third follow-up survey. The corresponding losses for women were − 0.10 (2.01), − 0.14 (2.28), and − 0.15 (2.60) kg/m^2^. Table 1 presents the sample’s baseline lipoprotein characteristics, and the average lipoprotein changes between the first, second and third follow-up visits.

**Table 1:** Baseline characteristics and mean changes from baseline, SI units

	Baseline	Difference from baseline^e^		
	Mean (SD)	Mean difference ± SE		
		1st follow-up	2nd follow-up	3rd follow-up
Males				
Triglycerides^a^	1.623 (1.330)	−0.100 ± 0.010	− 0.107 ± 0.016	− 0.132 ± 0.027
Log triglycerides	0.273 (0.544)	−0.054 ± 0.003	− 0.058 ± 0.006	− 0.075 ± 0.009
Apo B^b^	0.961 (0.287)	−0.045 ± 0.002	− 0.049 ± 0.004	− 0.054 ± 0.006
Non-HDL-cholesterol^a^	3.502 (1.171)	−0.237 ± 0.009	− 0.278 ± 0.015	− 0.321 ± 0.022
LDL-cholesterol direct^a^	2.975 (1.033)	−0.18 ± 0.007	− 0.21 ± 0.012	− 0.251 ± 0.019
LDL-cholesterol indirect^a^	2.797 (1.024)	−0.188 ± 0.007	− 0.234 ± 0.013	− 0.273 ± 0.018
sdLDL-cholesterol^a^	0.808 (0.45)	−0.064 ± 0.003	− 0.074 ± 0.005	− 0.08 ± 0.008
lbLDL-cholesterol^a^	2.17 (0.777)	−0.114 ± 0.005	− 0.139 ± 0.01	− 0.167 ± 0.014
%sdLDL-cholesterol^c^	23.23 (7.82)	−0.38 ± 0.07	− 0.43 ± 0.13	− 0.31 ± 0.20
LDL-particles^a^	1.322 (0.528)	−0.070 ± 0.005	− 0.069 ± 0.009	− 0.095 ± 0.013
Lp(a)^d^	5.192 (0.955)	−0.037 ± 0.006	− 0.041 ± 0.01	− 0.042 ± 0.015
Log Lp(a)	1.636 (0.183)	−0.008 ± 0.001	− 0.010 ± 0.002	− 0.011 ± 0.003
Females				
Triglycerides^a^	1.402 (0.923)	−0.052 ± 0.006	−0.072 ± 0.012	− 0.07 ± 0.013
Log triglycerides	0.273 (0.544)	−0.033 ± 0.003	−0.045 ± 0.005	− 0.050 ± 0.007
Apo B^b^	0.987 (0.281)	−0.029 ± 0.002	−0.036 ± 0.003	− 0.038 ± 0.006
Non-HDL-cholesterol^a^	3.63 (1.127)	−0.167 ± 0.007	−0.229 ± 0.014	− 0.27 ± 0.024
LDL-cholesterol direct^a^	3.177 (1.023)	−0.133 ± 0.006	−0.174 ± 0.012	− 0.212 ± 0.02
LDL-cholesterol indirect^a^	3.006 (1.019)	−0.152 ± 0.006	−0.208 ± 0.012	− 0.256 ± 0.02
sdLDL-cholesterol^a^	0.754 (0.393)	−0.042 ± 0.002	−0.05 ± 0.004	− 0.05 ± 0.007
lbLDL-cholesterol ^a^	2.426 (0.781)	−0.091 ± 0.005	−0.124 ± 0.009	− 0.156 ± 0.015
%sdLDL-cholesterol ^c^	0.714 (0.27)	−0.16 ± 0.05	−0.01 ± 0.08	0.19 ± 0.14
LDL-particles^a^	1.350 (0.513)	−0.048 ± 0.004	−0.066 ± 0.008	− 0.076 ± 0.013
Lp(a)^d^	6.145 (1.178)	−0.050 ± 0.006	−0.047 ± 0.011	− 0.05 ± 0.017
Log Lp(a)	1.803 (0.191)	−0.008 ± 0.001	−0.010 ± 0.002	− 0.011 ± 0.003

^a^ mmol/L; ^b^g/L; ^c^%; ^d^ μmol/L ^e^ Median time 0.8, 1.4, and 1.8 years for the first, second, and third follow-up visits, respectively

The age-adjusted regression analyses of Table 2 show the concordant relationships of ∆BMI vs. ∆triglycerides, ∆apo B, ∆nonHDL-cholesterol, ∆%sdLDL-cholesterol, and ∆LDL-particle number all were highly significant, a finding replicated in both men and women. Weight change was inversely related to ∆Lp(a) and weakly or unrelated to ∆lbLDL-cholesterol. The regression slopes for ∆LDL-cholesterol vs. ∆BMI were greater for the direct measurement than the calculated estimates of LDL-cholesterol. The effects of ∆BMI on ∆LDL-cholesterol and ∆lbLDL-cholesterol were generally less in men than women, whereas the effect of ∆BMI on ∆Lp(a) was greater in men.

**Table 2:** Regression analyses of age-adjusted ∆apoB-containing lipoproteins vs. ∆BMI over clinic visits. SI units

Dependent variable	Male		Female		Sex dif-ference (P)	Sample (N)
	Slope ± SE	Signif-icance	Slope ± SE	Signi-ficance		Male/female
∆Triglycerides^a^						
1st followup	0.0358 ± 0.0049	2.0 × 10^−13^	0.0304 ± 0.0029	< 10^−16^	0.33	14690/17977
2nd followup	0.0308 ± 0.0076	5.1 × 10^− 5^	0.033 ± 0.0053	3.4 × 10^−10^	0.77	5109/5893
3rd followup	0.0147 ± 0.0115	0.20	0.0315 ± 0.005	3.0 × 10^−10^	0.16	2334/2372
∆Log triglycerides						
1st followup	0.019 ± 0.002	< 10^−16^	0.019 ± 0.001	< 10^− 16^	0.96	14690/17977
2nd followup	0.024 ± 0.003	< 10^−16^	0.025 ± 0.002	< 10^−16^	0.89	5109/5893
3rd followup	0.020 ± 0.004	7.9 × 10^−8^	0.022 ± 0.003	2.7 × 10^−15^	0.57	2334/2372
∆Apo B^b^						
1st followup	0.0053 ± 0.0010	7.9 × 10^− 8^	0.0073 ± 0.0009	2.2 × 10^−16^	0.12	13384/16458
2nd followup	0.005 ± 0.0017	0.003	0.0072 ± 0.0015	9.6 × 10^−7^	0.29	4483/5231
3rd followup	0.0075 ± 0.0028	0.007	0.0067 ± 0.0023	0.003	0.85	1925/1973
∆nonHDL-cholesterol^a^						
1st followup	0.0238 ± 0.0041	7.9 × 10^−9^	0.0330 ± 0.0037	< 10^− 16^	0.07	12471/15149
2nd followup	0.0254 ± 0.0068	0.0002	0.0282 ± 0.0059	2.0 × 10^−6^	0.72	4323/4860
3rd followup	0.0235 ± 0.0089	0.008	0.0267 ± 0.009	0.003	0.81	1974/1979
∆LDL-cholesterol direct^a^						
1st followup	0.0128 ± 0.0034	0.0002	0.0232 ± 0.0031	1.2 × 10^−13^	0.02	14349/17625
2nd followup	0.0093 ± 0.0058	0.11	0.0202 ± 0.0051	7.5 × 10^−5^	0.14	4985/5792
3rd followup	0.0134 ± 0.0078	0.09	0.0235 ± 0.0075	0.002	0.34	2287/2331
∆LDL-cholesterol indirect^a^						
1st followup	0.0084 ± 0.0034	0.01	0.0186 ± 0.0031	1.6 × 10^−9^	0.02	14238/17455
2nd followup	0.0048 ± 0.0059	0.41	0.0134 ± 0.0051	0.008	0.22	4954/5700
3rd followup	0.0139 ± 0.0077	0.07	0.0198 ± 0.0076	0.009	0.56	2282/2307
∆sdLDL-cholesterol^a^						
1st followup	0.0125 ± 0.0015	2.2 × 10^−16^	0.0128 ± 0.0012	< 10^− 16^	0.85	13668/16875
2nd followup	0.0109 ± 0.0025	8.2 × 10^−6^	0.0144 ± 0.0019	8.4 × 10^−15^	0.21	4721/5524
3rd followup	0.0121 ± 0.0033	0.0003	0.0123 ± 0.0027	4.6 × 10^−6^	0.92	2185/2235
∆lbLDL-cholesterol ^a^						
1st followup	0.0007 ± 0.0026	0.78	0.0098 ± 0.0024	3.9 × 10^−5^	0.008	13518/16727
2nd followup	−0.0005 ± 0.0044	0.90	0.0055 ± 0.0039	0.16	0.27	4680/5475
3rd followup	0.0021 ± 0.0059	0.72	0.0114 ± 0.0057	0.05	0.25	2174/2210
∆%sdLDL-cholesterol^c^						
1st followup	0.296 ± 0.035	< 10^−16^	0.221 ± 0.023	< 10^− 16^	0.06	10605/13757
2nd followup	0.294 ± 0.06	8.2 × 10^−7^	0.259 ± 0.038	6.4 × 10^−12^	0.63	3429/4229
3rd followup	0.389 ± 0.098	6.9 × 10^−5^	0.196 ± 0.056	0.0004	0.07	1438/1522
∆LDL-particle number^a^						
1st followup	0.0128 ± 0.0024	2.1 × 10^− 7^	0.0114 ± 0.0022	3.2 × 10^− 7^	0.69	7408/9025
2nd followup	0.0100 ± 0.0036	0.005	0.0109 ± 0.0034	0.002	0.81	2600/3004
3rd followup	0.0122 ± 0.0046	0.008	0.0117 ± 0.0048	0.02	0.92	1177/1289
∆Lp(a)^d^						
1st followup	−0.0206 ± 0.0064	0.001	−0.0010 ± 0.0030	0.75	0.009	13126/16094
2nd followup	−0.0257 ± 0.0087	0.003	−0.0121 ± 0.0047	0.01	0.15	4587/5277
3rd followup	−0.029 ± 0.0094	0.002	−0.0092 ± 0.0064	0.15	0.19	2138/2169
∆Log Lp(a)^d^						
1st followup	−0.002 ± 0.001	4.7 × 10^−5^	0.000 ± 0.000	0.94	0.003	13113/16077
2nd followup	−0.004 ± 0.001	8.6 × 10^−6^	− 0.002 ± 0.001	0.02	0.06	4586/5273
3rd followup	−0.004 ± 0.001	0.004	−0.002 ± 0.001	0.11	0.20	2138/2169

^a^ ∆mmol/L per ∆kg/m^2^; ^b^ ∆g/L per ∆kg/m^2^; ^c^∆% per ∆kg/m^2^; ^d^∆μmol/L per ∆kg/m^2^

Table 3 tests whether the aforementioned associations can be simply attributed to ∆triglycerides and ∆LDL-cholesterol. When sexes were combined and adjusted for age, sex, ∆triglycerides and ∆LDL-cholesterol, ∆BMI: 1) gained a strongly significant inverse relationship with ∆lbLDL, 2) retained the all of its significant residual association with ∆Lp(a) and the majority of its association with ∆%sdLDL-cholesterol, and 3) maintained significant but much reduced associations with ∆apo B, ∆nonHDL-cholesterol, ∆LDL-particles, and ∆sdLDL-cholesterol. The analyses were repeated separately in males and females for ∆lbLDL-cholesterol due to its significant sex differences of Table 2. In males and females separately, ∆BMI was significantly associated with both ∆lbLDL-cholesterol (males: − 0.0047 ± 0.0009, *P* = 3.6 × 10^− 7^; females: − 0.0038 ± 0.0007 ∆mmol/L per ∆kg/m^2^, *P* = 3.4 × 10^− 8^) when adjusted. Similar results were obtained when the preceding analyses used ∆Log triglycerides rather than ∆triglycerides as a covariate (analyses not displayed).

**Table 3:** Regression analyses of age- and sex-adjusted ∆apoB-containing lipoproteins vs. ∆BMI over clinic visits, SI units

	Adjusted for age and sex only		Additional adjustment for ∆triglycerides and ∆LDL-cholesterol		Sample (N)
Dependent variable	Slope ± SE	Significance	Slope ± SE	Significance	
∆Apo B^b^					
1st followup	0.0064 ± 0.0007	< 10^− 16^	0.001 ± 0.0003	0.0008	29342
2nd followup	0.0063 ± 0.0011	1.9 × 10^−8^	0.0016 ± 0.0005	0.0006	9642
3rd followup	0.007 ± 0.0017	5.8 × 10^−5^	0.001 ± 0.0007	0.16	3877
∆nonHDL-cholesterol ^a^					
1st followup	0.0289 ± 0.0027	< 10^−16^	0.0019 ± 0.0009	0.03	27613
2nd followup	0.027 ± 0.0045	1.5 × 10^−9^	0.0058 ± 0.0015	0.0001	9180
3rd followup	0.0252 ± 0.0063	6.8 × 10^−5^	0.0088 ± 0.0022	6.3 × 10^− 5^	3952
∆sdLDL-cholesterol ^a^					
1st followup	0.0008 ± 0.0001	< 10^−16^	0.0001 ± 0.0000	1.6 × 10^−11^	29988
2nd followup	0.0007 ± 0.0001	< 10^−16^	0.0002 ± 0.0001	1.5 × 10^−11^	10164
3rd followup	0.0006 ± 0.0002	6.6 × 10^−9^	0.0002 ± 0.0001	0.0003	4396
∆lbLDL-cholesterol ^a^					
1st followup	0.0059 ± 0.0018	0.0009	−0.0041 ± 0.0006	3.4 × 10^−13^	30056
2nd followup	0.0029 ± 0.0029	0.33	−0.0059 ± 0.0009	3.8 × 10^−11^	10124
3rd followup	0.0073 ± 0.0041	0.08	−0.0052 ± 0.0012	3.2 × 10^−15^	4376
∆%sdLDL-cholesterol^c^					
1st followup	0.254 ± 0.02	< 10^−16^	0.151 ± 0.018	< 10^−16^	24274
2nd followup	0.274 ± 0.034	4.4 × 10^−16^	0.186 ± 0.03	3.8 × 10^−10^	7633
3rd followup	0.27 ± 0.053	2.9 × 10^−7^	0.218 ± 0.049	8.2 × 10^−6^	2952
∆LDL-particle number^a^					
1st followup	0.0121 ± 0.0017	2.9 × 10^−13^	0.0032 ± 0.0010	0.001	16135
2nd followup	0.0105 ± 0.0025	2.6 × 10^−5^	0.0039 ± 0.0015	0.01	5554
3rd followup	0.0119 ± 0.0033	0.0003	0.0037 ± 0.0022	0.10	2452
∆Lp(a)^d^					
1st followup	−0.0059 ± 0.0021	0.005	−0.0091 ± 0.0021	1.2 × 10^−5^	28096
2nd followup	−0.0163 ± 0.0033	7.8 × 10^−7^	− 0.018 ± 0.0033	5.3 × 10^−8^	9673
3rd followup	−0.0142 ± 0.0045	0.002	−0.0155 ± 0.0045	0.0006	4264
∆Log Lp(a)					
1st followup	−0.001 ± 0	0.005	−0.002 ± 0	9.0 × 10^−6^	28096
2nd followup	−0.003 ± 0.001	2.9 × 10^− 6^	−0.003 ± 0.001	2.2 × 10^−7^	9673
3rd followup	−0.003 ± 0.001	0.002	−0.003 ± 0.001	0.0006	4264
∆Triglycerides^a^					
1st followup	0.0328 ± 0.0027	< 10^−16^			32674
2nd followup	0.032 ± 0.0045	7.5 × 10^−13^			11001
3rd followup	0.0241 ± 0.006	6.1 × 10^−5^			4706
∆LDL-cholesterol^a^					
1st followup	0.0186 ± 0.0023	6.7 × 10^−16^			31981
2nd followup	0.0155 ± 0.0038	5.3 × 10^−5^			10776
3rd followup	0.019 ± 0.0054	0.0004			4618

^a^ ∆mmol/L per ∆kg/m^2^; ^b^ ∆g/L per ∆kg/m^2^; ^c^∆% per ∆kg/m^2^; ^d^∆μmol/L per ∆kg/m^2^

## Discussion

These analyses examine the associations between ∆BMI and ∆apoB containing lipoproteins before and after adjustment for ∆triglycerides and ∆LDL-cholesterol. This was done to assess whether these emerging risk factors provide additional information on the benefits and hazards of body weight vis-á-vis traditional lipid measurements. Table 2 showed that ∆BMI had a stronger effect (larger regression slope) on ∆LDL-cholesterol measured directly than on ∆LDL-cholesterol estimated indirectly by the Friedewald equation. Although the Friedewald estimates were strongly correlated with the direct measurement (*r* = 0.98), they are reported to underestimate the direct measurement by about 5% with increasing inaccuracy with increasing triglyceride concentrations [4]. In addition, body weight change was associated with significant changes in apoB, nonHDL-cholesterol, LDL-particle, sdLDL-cholesterol, %sdLDL-cholesterol concentrations, and Lp(a) but not lbLDL-concentrations concentrations. The associations were unaffected by sex for ∆triglycerides, ∆apoB, ∆nonHDL-cholesterol, and ∆LDL-particle concentrations, were somewhat stronger in women than men for ∆LDL-cholesterol and ∆lbLDL-cholesterol, and stronger in men than women for ∆Lp(a).

The effects of weight loss on LDL are well established. Meta-analyses of weight loss studies estimate that a 16 kg or 16% average weight loss produces a 0.78 mmol/L (30 mg/dL) average reduction in total cholesterol due to equal reductions in LDL-cholesterol and VLDL-cholesterol, and an average LDL-cholesterol reduction of 0.021 mmol/L (0.8 mg/dL) per kg lost [30]. Equal effects on LDL- and VLDL-cholesterol support the use of nonHDL-cholesterol in the analysis of weight loss. Meta-analyses have also shown significant reductions in apo B concentrations in hypoenergic diets producing 6–12% weight loss [31]. Tables 2 and 3 showed that ∆apo B, ∆nonHDL-cholesterol, ∆LDL-particles, and ∆%sdLDL all showed significantly stronger (i.e, more significant) relationships to ∆BMI than the traditional ∆LDL-cholesterol concentrations, suggesting potentially even greater health benefits of weight management. Their relationships with ∆BMI remained significant when adjusted for ∆triglycerides and ∆LDL-cholesterol.

As expected, ∆triglycerides and ∆LDL-cholesterol were concordantly related with ∆BMI. High triglyceride levels associated with abdominal obesity are due to enhanced secretion (accounting for 20% of plasma triglyceride concentrations) and impaired clearance of triglyceride-rich lipoproteins (accounting for 50%) [32]. Total body fat and liver fat content largely determine the amount of triglyceride secreted by the liver, mostly in the form of large triglyceride rich VLDL1 particles, but also VLDL2. The VLDL-triglycerides are hydrolyzed by lipoprotein lipase on the on the luminal surfaces of muscle and adipose tissue, releasing fatty acids for energy production or storage. The impaired clearance is largely due to increased levels of apo C-III, an inhibitor of hepatic and lipoprotein lipase that also impairs VLDL clearance by interfering with the binding of apoB and apoE to hepatic receptors [32–34].

Unlike other apo B-containing lipoproteins, changes in Lp(a) were inversely related to BMI change, particularly in men, and the association was somewhat strengthened when adjusted for changes in triglycerides and LDL-cholesterol. Their inverse relationship has been noted by others [35–37], and although other studies report little effect of weight loss on Lp(a) concentrations [38], they involve substantially fewer subjects than reported here.

### LDL-subclasses

Historically, LDL heterogeneity were categorized in terms of the relative concentrations of large and small LDL as phenotypes A (preponderance of large LDL) and phenotype B (preponderance of small LDL) or the LDL-peak particle diameter (estimated diameter of the mean or mode of LDL particle distribution) [14], which corresponds most closely with the %sdLDL of the present analyses. This parameterization is most appropriate for metabolic processes that alter the equilibrium distribution of large and small LDL, e.g., if body weight increases triglycerides concentrations, which in turn shifts the precursor-product relationship from lbLDL (1.044–1.063 g/ml density) to sdLDL (1.019–1.043 g/ml density) due to increased cholesteryl ester triglyceride exchange, then adjustment for ∆triglyceride concentrations should mostly eliminate the association between ∆BMI and ∆%sdLDL, which was not observed. In fact, Table 3 suggests the majority of the effect of ∆BMI on ∆%sdLDL was independent of triglycerides and LDL-cholesterol concentrations. This agrees with compartmental models that exclude the possibility of sdLDL being produced exclusively from the delipidation of lbLDL [39].

At least some of the concordance between ∆sdLDL and ∆BMI was attributable to ∆triglycerides, albeit not the majority. sdLDL particles are derived from multiple sources, providing multiple pathways by which ∆weight-mediated ∆triglycerides could affect ∆sdLDL concentrations. Some sdLDL is derived directly from VLDL and IDL independently of lbLDL, some from the delipidation of lbLDL, and some secreted directly by the liver [39]. High triglycerides concentrations are associated with greater hepatic secretion of sdLDL particles than lbLDL particles, a greater number of VLDL particles being converted to sdLDL density than catabolized, a greater fraction of lbLDL delipidated to sdLDL, and slow clearance of sdLDL due to its greater apo CIII content [40].

The lack of concordance between ∆lbLDL and ∆BMI may be due to two counterbalancing effects of triglyceride enrich lipoproteins: lower concentrations resulting in reduced cholesteryl ester-triglyceride exchange (an lbLDL increasing effect) and reduced availability of lbLDL-precursors (an lbLDL decreasing effect), while higher concentrations having the opposite consequences. Specifically, lower plasma triglyceride concentration with weight loss may limit the cholesteryl ester transfer protein (CETP)-mediated exchange of VLDL-triglycerides for LDL-cholesteryl esters leading to an accumulation of lbLDL-cholesterol in parallel with increasing HDL-cholesterol concentrations [39]. The CETP-inhibitor torcetrapib has been shown to produce parallel increases large LDL and HDL-cholesterol [41]. Fewer available precursors and less exchange may result in no net lbLDL increase.

Differences in the metabolic regulation of sdLDL and lbLDL are also presumed to explain the LDL-cholesterol differences for weight loss by traditional low-fat diets vs. high fat ketogenic diets. Both reduce plasma concentrations of small dense LDL. However, weight loss achieved by very-low-carbohydrate ketogenic diets significantly increase LDL-cholesterol and decreased triglycerides in association with weight loss compared to a traditional low-fat diet (4.6 mg/dL greater LDL-cholesterol increase and 15.9 mg/dL greater triglyceride decrease for 0.91 kg greater average weight loss) [42]. This presumably relates to the higher saturated fat intake of ketogenic diets, which is estimated to increase LDL-cholesterol by 0.8 to 1.6 mg/dL for each 1% increase in energy from saturated fat [43]. Increased saturated fat intake is reported to affect the larger more buoyant LDL in the context of limiting carbohydrate intake [44].

In addition to identifying differences in their metabolic regulation, the subclass specific associations between ∆BMI and ∆LDL-cholesterol are important because sdLDL and lbLDL show very different associations with CVD risk [45]. Compared to sdLDL, lbLDL are less readily oxidized in vitro [46], have less affinity proteoglycans in the arterial wall [47], contain a lower percentage of glycated apo B [48], and are overall less susceptible to glycation [49]. Prospective cohort and case-control studies show greater ASCVD risk for sdLDL and not lbLDL [15, 50, 51], although as in the case of its BMI association, the increased ASCVD risk of sdLDL is not necessarily independent of plasma triglycerides.

### Caveats and limitations

An important strength of this large clinical database analysis is the opportunity to assess the magnitude of weight-related changes in a variety of atherogenic lipoprotein particles in the context of actual clinical practice. However, although the population generally reflects the outpatient primary care population of thousands of diverse medical practices, it is not feasible to precisely define the clinical characteristics of the specific study population. Important examples include the lack of more-extensive information on lifestyle (such as dietary intake, physical activity, or causes of weight change), medical treatment (such as medications) and general health status. This limits the ability to statistically adjust for different causes of weight change, which could potentially modify the statistical relationship between changes in weight and various atherogenic particles. To avoid confounding by the presence or absence of lipid-lowering medication use, we excluded individuals known to take such medications. Nevertheless, in some cases the absence of lipid-lowering medications may have been due to nonresponse. Waist circumference, an index of intra-abdominal fat that likely has a more direct relationship to altered lipoprotein metabolism in the obese state [52], was not available. The associations reported here do not prove causality, although the causal relationship between weight loss and apoB-containing lipoproteins is firmly established by others [30, 31]. The sample was not obtained under any predefined sampling strategy. However, the sample is likely not atypical of the conditions encountered under usual practice and therefore provide realistic expectations of the improvement or decline associated with changing weight in the clinical setting. Whereas most prior reports on LDL-subfractions in relation to weight loss are from small studies, the over 30,000 subjects reported here provides precise estimates for LDL-subfractions known to be important for their difference in metabolism, functionality, and cardiovascular disease risk.

## Conclusion

The emerging apo B-containing risk factors examined in this report show associations with weight change beyond those explained by the more-traditional measurements of triglyceride and LDL-cholesterol.

## Additional file

Additional file 1: Supplemental Data File. (DOCX 82 kb)

## Data Availability

The datasets analyzed in this report may be available from the corresponding author on reasonable request.

## Abbreviations

%sdLDL: percent of LDL-cholesterol carried on small dense low-density lipoproteins
BMI: Body mass index
HDL: High density lipoproteins
lbLDL: Large buoyant low-density lipoproteins
LDL: Low-density lipoproteins
Lp(a): Lipoprotein(a)
sdLDL: small dense low-density lipoproteins
VLDL: Very low density lipoproteins
