# Supplementary material for: Effects of weight change on apolipoprotein B-containing emerging atherosclerotic cardiovascular disease (ASCVD) risk factors
Source: Lipids Health Dis. 2019 Jul 17;18:154. doi: 10.1186/s12944-019-1094-4 (PMC6636168; doi:10.1186/s12944-019-1094-4)
Supplement: Supplementary file 1 — Supplemental Data File. (DOCX 82 kb) [file 12944_2019_1094_MOESM1_ESM.docx]

Effects of weight change on apolipoprotein B-containing emerging atherosclerotic cardiovascular disease (ASCVD) risk factors.

Supplementary material

Michael Dansinger, MD^1,2,4^

Paul T Williams, PhD^1^

H Robert Superko, MD^1^

Ernst J. Schaefer, MD^1,3^

^1^Boston Heart Diagnostics

175 Crossing Boulevard, Suite 100

Framingham, MA 01702

^2^Tufts Medical Center,

800 Washington St.

Boston MA 02111.

^3^Cardiovascular Nutrition Laboratory,

USDA Human Nutrition Research Center at Tufts University,

711 Washington St. Boston MA 02111

^4^To whom all correspondence should be sent

[MDansinger@BostonHeartDx.com](mailto:MDansinger@BostonHeartDx.com)

| Table S1. Baseline characteristics and mean changes from baseline, conventional units | | | | |
| --- | --- | --- | --- | --- |
|  | Baseline | Difference from baseline | | |
|  | Mean (SD) | Mean difference±SE | | |
|  |  | 1^st^ follow-up | 2^nd^ follow-up | 3^rd^ follow-up |
| Males |  |  |  |  |
| Triglycerides* | 137.57 (112.69) | -8.49±0.83 | -9.11±1.38 | -11.15±2.30 |
| Log triglycerides | 4.756 (0.544) | -0.054±0.003 | -0.058±0.006 | -0.075±0.009 |
| Apo B* | 96.06 (28.71) | -4.48±0.20 | -4.87±0.37 | -5.44±0.56 |
| Non-HDL-cholesterol* | 135.21 (45.21) | -9.14±0.33 | -10.75±0.57 | -12.38±0.86 |
| LDL-cholesterol direct* | 114.86 (39.88) | -6.96±0.27 | -8.12±0.48 | -9.70±0.72 |
| LDL-cholesterol indirect* | 108.00 (39.54) | -7.26±0.27 | -9.04±0.49 | -10.55±0.71 |
| sdLDL-cholesterol* | 31.19 (17.37) | -2.49±0.12 | -2.85±0.21 | -3.10±0.31 |
| lbLDL-cholesterol * | 83.77 (30.01) | -4.39±0.20 | -5.35±0.37 | -6.46±0.55 |
| %sdLDL (LDL-cholesterol carried on small LDL) | 23.23 (7.82) | -0.38±0.07 | -0.43±0.13 | -0.31±0.20 |
| LDL-particles^†^ | 1321.93 (528.16) | -69.97±4.88 | -69.22±8.65 | -94.57±12.53 |
| Lp(a)* | 145.44 (26.75) | -1.04±0.16 | -1.14±0.28 | -1.19±0.42 |
| Log Lp(a)* | 4.963 (0.183) | -0.008±0.001 | -0.010±0.002 | -0.011±0.003 |
|  |  |  |  |  |
| Females |  |  |  |  |
| Triglycerides* | 118.82 (78.18) | -4.38±0.50 | -6.11±1.02 | -5.97±1.12 |
| Log triglycerides | 4.642 (0.498) | -0.033±0.003 | -0.045±0.005 | -0.050±0.007 |
| Apo B* | 98.65 (28.13) | -2.85±0.18 | -3.64±0.33 | -3.84±0.57 |
| Non-HDL-cholesterol* | 140.14 (43.53) | -6.46±0.28 | -8.84±0.53 | -10.42±0.91 |
| LDL-cholesterol direct* | 122.65 (39.50) | -5.12±0.24 | -6.73±0.45 | -8.19±0.77 |
| LDL-cholesterol indirect* | 116.07 (39.36) | -5.88±0.24 | -8.03±0.45 | -9.87±0.77 |
| sdLDL-cholesterol* | 29.13 (15.18) | -1.63±0.09 | -1.92±0.17 | -1.94±0.27 |
| lbLDL-cholesterol* | 93.65 (30.15) | -3.53±0.18 | -4.77±0.35 | -6.04±0.58 |
| %sdLDL (LDL-cholesterol carried on small LDL) | 27.55 (10.43) | -0.16±0.05 | -0.01±0.08 | 0.19±0.14 |
| LDL-particles^†^ | 1350.18 (512.91) | -47.91±4.25 | -66.04±7.90 | -76.44±13.00 |
| Lp(a)* | 172.13 (33.00) | -1.40±0.17 | -1.32±0.30 | -1.40±0.47 |
| Log Lp(a)* | 5.130 (0.191) | -0.008±0.001 | -0.008±0.002 | -0.009±0.003 |
| * mg/dL; ^†^ nmol/L; | | | | |

| Table S2. Regression analyses of age-adjusted ∆apoB-containing lipoproteins vs. ∆BMI over clinic visits, conventional units. | | | | | | |
| --- | --- | --- | --- | --- | --- | --- |
|  | Male | | Female | | Sex dif-ference  (P) | Sample (N) |
| Dependent variable | Slope±SE | Signif-icance | Slope±SE | Signi-ficance |  | Male/female |
| ∆Triglycerides* | | | | | | |
| 1st followup | 3.032±0.413 | 2.0x10^-13^ | 2.574±0.246 | <10^-16^ | 0.33 | 14690/17977 |
| 2nd followup | 2.606±0.644 | 5.1x10^-5^ | 2.799±0.446 | 3.4x10^-10^ | 0.77 | 5109/5893 |
| 3rd followup | 1.249±0.972 | 0.20 | 2.67±0.424 | 3.0x10^-10^ | 0.16 | 2334/2372 |
| ∆Log triglycerides | | | | | | |
| 1st followup | 0.019±0.002 | <10^-16^ | 0.019±0.001 | <10^-16^ | 0.96 | 14690/17977 |
| 2nd followup | 0.024±0.003 | <10^-16^ | 0.025±0.002 | <10^-16^ | 0.89 | 5109/5893 |
| 3rd followup | 0.020±0.004 | 7.9x10^-8^ | 0.022±0.003 | 2.7x10^-15^ | 0.57 | 2334/2372 |
| ∆Apo B* | | | | | | |
| 1st followup | 0.533±0.099 | 7.9x10^-8^ | 0.729±0.089 | 2.2x10^-16^ | 0.12 | 13384/16458 |
| 2nd followup | 0.504±0.17 | 0.003 | 0.723±0.148 | 9.6x10^-7^ | 0.29 | 4483/5231 |
| 3rd followup | 0.748±0.276 | 0.007 | 0.673±0.227 | 0.003 | 0.85 | 1925/1973 |
| ∆nonHDL-cholesterol* | | | | | | |
| 1st followup | 0.917±0.159 | 7.9x10^-9^ | 1.275±0.141 | 0<10^-16^ | 0.07 | 12471/15149 |
| 2nd followup | 0.982±0.262 | 0.0002 | 1.09±0.229 | 2.0x10^-6^ | 0.72 | 4323/4860 |
| 3rd followup | 0.907±0.344 | 0.008 | 1.03±0.347 | 0.003 | 0.81 | 1974/1979 |
| ∆LDL-cholesterol direct* | | | | | | |
| 1st followup | 0.496±0.133 | 0.0002 | 0.894±0.12 | 1.2x10^-13^ | 0.02 | 14349/17625 |
| 2nd followup | 0.359±0.224 | 0.11 | 0.778±0.196 | 7.5x10^-5^ | 0.14 | 4985/5792 |
| 3rd followup | 0.516±0.303 | 0.09 | 0.906±0.29 | 0.002 | 0.34 | 2287/2331 |
| ∆LDL-cholesterol indirect* | | | | | | |
| 1st followup | 0.326±0.132 | 0.01 | 0.72±0.119 | 1.6x10^-9^ | 0.02 | 14238/17455 |
| 2nd followup | 0.187±0.227 | 0.41 | 0.519±0.195 | 0.008 | 0.22 | 4954/5700 |
| 3rd followup | 0.538±0.299 | 0.07 | 0.764±0.294 | 0.009 | 0.56 | 2282/2307 |
| ∆sdLDL-cholesterol* | | | | | | |
| 1st followup | 0.481±0.058 | 2.2x10^-16^ | 0.493±0.045 | <10^-16^ | 0.85 | 13668/16875 |
| 2nd followup | 0.422±0.095 | 8.2x10^-6^ | 0.557±0.072 | 8.4x10^-15^ | 0.21 | 4721/5524 |
| 3rd followup | 0.469±0.128 | 0.0003 | 0.475±0.104 | 4.6x10^-6^ | 0.92 | 2185/2235 |
| ∆lbLDL-cholesterol * | | | | | | |
| 1st followup | 0.028±0.101 | 0.78 | 0.378±0.092 | 3.9x10^-5^ | 0.008 | 13518/16727 |
| 2nd followup | -0.02±0.171 | 0.90 | 0.211±0.15 | 0.16 | 0.27 | 4680/5475 |
| 3rd followup | 0.083±0.227 | 0.72 | 0.442±0.222 | 0.05 | 0.25 | 2174/2210 |
| ∆%sdLDL-cholesterol ^‡^ | | | | | | |
| 1st followup | 0.296±0.035 | <10^-16^ | 0.221±0.023 | <10^-16^ | 0.06 | 10605/13757 |
| 2nd followup | 0.294±0.06 | 8.2x10^-7^ | 0.259±0.038 | 6.4x10^-12^ | 0.63 | 3429/4229 |
| 3rd followup | 0.389±0.098 | 6.9x10^-5^ | 0.196±0.056 | 0.0004 | 0.07 | 1438/1522 |
| ∆LDL-particle number^†^ | | | | | | |
| 1st followup | 12.844±2.474 | 2.1x10^-7^ | 11.377±2.225 | 3.2x10^-7^ | 0.69 | 7408/9025 |
| 2nd followup | 9.99±3.562 | 0.005 | 10.948±3.485 | 0.002 | 0.81 | 2600/3004 |
| 3rd followup | 12.225±4.579 | 0.008 | 11.678±4.831 | 0.02 | 0.92 | 1177/1289 |
| ∆Lp(a)* | | | | | | |
| 1st followup | -0.577±0.178 | 0.001 | -0.027±0.083 | 0.75 | 0.009 | 13126/16094 |
| 2nd followup | -0.719±0.245 | 0.003 | -0.34±0.132 | 0.01 | 0.15 | 4587/5277 |
| 3rd followup | -0.811±0.263 | 0.002 | -0.259±0.179 | 0.15 | 0.19 | 2138/2169 |
| ∆Log Lp(a)* | | | | | | |
| 1st followup | -0.002±0.001 | 4.7x10^-5^ | 0.00±0.00 | 0.94 | 0.003 | 13113/16077 |
| 2nd followup | -0.004±0.001 | 8.6x10^-6^ | -0.002±0.001 | 0.02 | 0.06 | 4586/5273 |
| 3rd followup | -0.004±0.001 | 0.004 | -0.002±0.001 | 0.11 | 0.20 | 2138/2169 |
| * ∆mg/dL per ∆kg/m^2^; ^†^ ∆nmol/L per ∆kg/m^2^; ^‡^∆% per ∆kg/m^2^ | | | | | | |

| Table S3. Regression analyses of age- and sex-adjusted ∆apoB-containing lipoproteins vs. ∆BMI over clinic visits, conventional units | | | | | | |
| --- | --- | --- | --- | --- | --- | --- |
|  | Adjusted for age and sex only | | Additional adjustment for ∆triglycerides and ∆LDL-cholesterol | |  | Sample (N) |
| Dependent variable | Slope±SE | Significance | Slope±SE | Significance |  |  |
| ∆Apo B* | | | | | | |
| 1st followup | 0.643±0.066 | <10^-16^ | 0.095±0.028 | 0.0008 |  | 29342 |
| 2nd followup | 0.627±0.111 | 1.9x10^-8^ | 0.158±0.046 | 0.0006 |  | 9642 |
| 3rd followup | 0.701±0.174 | 5.8x10^-5^ | 0.097±0.069 | 0.16 |  | 3877 |
| ∆nonHDL* | | | | | | |
| 1st followup | 1.116±0.105 | <10^-16^ | 0.074±0.033 | 0.03 |  | 27613 |
| 2nd followup | 1.042±0.173 | 1.5x10^-9^ | 0.224±0.058 | 0.0001 |  | 9180 |
| 3rd followup | 0.972±0.244 | 6.8x10^-5^ | 0.338±0.084 | 6.3x10^-5^ |  | 3952 |
| ∆sdLDL-cholesterol * | | | | | | |
| 1st followup | 0.488±0.036 | <10^-16^ | 0.144±0.021 | 1.6x10^-11^ |  | 29988 |
| 2nd followup | 0.499±0.058 | <10^-16^ | 0.231±0.034 | 1.5x10^-11^ |  | 10164 |
| 3rd followup | 0.472±0.081 | 6.6x10^-9^ | 0.168±0.047 | 0.0003 |  | 4396 |
| ∆lbLDL-cholesterol * | | | | | | |
| 1st followup | 0.227±0.068 | 0.0009 | -0.159±0.022 | 3.4x10^-13^ |  | 30056 |
| 2nd followup | 0.111±0.113 | 0.33 | -0.226±0.034 | 3.8x10^-11^ |  | 10124 |
| 3rd followup | 0.28±0.159 | 0.08 | -0.199±0.048 | 3.2x10^-15^ |  | 4376 |
| ∆%sd LDL-cholesterol ^‡^ | | | | | | |
| 1st followup | 0.254±0.02 | <10^-16^ | 0.151±0.018 | <10^-16^ |  | 24274 |
| 2nd followup | 0.274±0.034 | 4.4x10^-16^ | 0.186±0.03 | 3.8x10^-10^ |  | 7633 |
| 3rd followup | 0.27±0.053 | 2.9x10^-7^ | 0.218±0.049 | 8.2x10^-6^ |  | 2952 |
| ∆LDL-particle number† | | | | | | |
| 1st followup | 12.078±1.654 | 2.9x10^-13^ | 3.245±1.009 | 0.001 |  | 16135 |
| 2nd followup | 10.472±2.49 | 2.6x10^-5^ | 3.926±1.547 | 0.01 |  | 5554 |
| 3rd followup | 11.943±3.336 | 0.0003 | 3.665±2.206 | 0.10 |  | 2452 |
| ∆Lp(a)* |  |  |  |  |  |  |
| 1st followup | -0.164±0.058 | 0.005 | -0.256±0.059 | 1.2 x10^-5^ |  | 28096 |
| 2nd followup | -0.457±0.092 | 7.8x10^-7^ | -0.504±0.093 | 5.3x10^-8^ |  | 9673 |
| 3rd followup | -0.398±0.126 | 0.002 | -0.434±0.126 | 0.0006 |  | 4264 |
| ∆Log Lp(a) |  |  |  |  |  |  |
| 1st followup | -0.001±0 | 0.005 | -0.002±0 | 9.0 x10^-6^ |  | 28096 |
| 2nd followup | -0.003±0.001 | 2.9x10^-6^ | -0.003±0.001 | 2.2x10^-7^ |  | 9673 |
| 3rd followup | -0.003±0.001 | 0.002 | -0.003±0.001 | 0.0006 |  | 4264 |
| ∆Triglycerides* |  |  |  |  |  |  |
| 1st followup | 2.782±0.23 | <10^-16^ |  |  |  | 32674 |
| 2nd followup | 2.716±0.379 | 7.5x10^-13^ |  |  |  | 11001 |
| 3rd followup | 2.04±0.508 | 6.1x10^-5^ |  |  |  | 4706 |
| ∆LDL-cholesterol* |  |  |  |  |  |  |
| 1st followup | 0.719±0.089 | 6.7 x10^-16^ |  |  |  | 31981 |
| 2nd followup | 0.597±0.148 | 5.3 x10^-5^ |  |  |  | 10776 |
| 3rd followup | 0.734±0.209 | 0.0004 |  |  |  | 4618 |
| * ∆mg/dL per ∆kg/m^2^; ^†^ ∆nmol/L per ∆kg/m^2^; ^‡^∆% per ∆kg/m^2^ | | | | | | |
